# Thirteen-year viral suppression and immunologic recovery of LPV/r-based regimens in pediatric HIV treatment: a multicenter cohort study in resource-constrained settings of China

**DOI:** 10.3389/fmed.2023.1313734

**Published:** 2023-12-22

**Authors:** Xiaojie Lao, Hanxi Zhang, Liting Yan, Hongxin Zhao, Qingxia Zhao, Hongyan Lu, Yuewu Chen, Huiqin Li, Jinfeng Chen, Fuxiu Ye, Fengting Yu, Qing Xiao, Qun Li, Xuelei Liang, Xiaojie Yang, Chang Yan, Fujie Zhang

**Affiliations:** ^1^Department of Infectious Disease, Beijing Ditan Hospital, Capital Medical University, Beijing, China; ^2^WHO Collaborating Centre for Comprehensive Management of HIV Treatment and Care, Beijing Ditan Hospital Capital Medical University, Beijing, China; ^3^Department of Infectious Disease, Sichuan Provincial People's Hospital, University of Electronic Science and Technology of China, Chengdu, China; ^4^Department of Infectious Disease, The Sixth People's Hospital of Zhengzhou, Zhengzhou, China; ^5^Department of Infectious Disease, Guangxi Zhuang Autonomous Region Center for Disease Control and Prevention, Nanning, China; ^6^Department of Infectious Disease, Shangcai Center for Disease Control and Prevention of Henan Province, Shangcai, China; ^7^AIDS Care Center, Yunnan Provincial Hospital of Infectious Disease, Kunming, China; ^8^Center for Infectious Diseases, Guangzhou Eighth People's Hospital, Guangzhou Medical University, Guangzhou, China; ^9^Department of Infectious Disease, The Second People's Hospital of Yining, Xinjiang, China

**Keywords:** HIV, lopinavir/ritonavir, LPV/r, children, effectiveness, safety, antiretroviral therapy, ART

## Abstract

**Background:**

Antiretroviral Therapy (ART) in children remains challenging due to resource-constrained settings. We conducted a 13-year, prospective, multicenter cohort study on the effectiveness and safety of LPV/r-based regimens in ART-naive and ART-experienced children.

**Methods:**

From January 2008 to May 2021, children living with HIV-1 were recruited with LPV/r-based regimens from 8 clinical research sites in 6 provinces in China. Effectiveness outcomes were virologic failure (defined as at least two consecutive measurements of VL > 200 copies/mL after 6 months of ART) and immune response (defined as CD4% recovered to more than 25% after 12 months of treatment). The safety outcomes were treatment-related grade 2–4 adverse events and abnormal laboratory test results.

**Results:**

A total of 345 ART-naïve children and 113 ART-experienced children were included in this cohort study. The median follow-up time was 7.3 (IQR 5.5–10.5) years. The incidence density of virologic failure was 4.1 (95% CI 3.3–4.9) per 100 person-years in ART-naïve children and 5.0 (95% CI 3.5–6.5) per 100 person-years in ART-experienced children. Kaplan Meyer (KM) curve analysis showed children with ART experience were at a higher risk of virologic failure (*p* < 0.05). The risk factors of virologic failure in ART-naïve children were clinic setting in rural hospitals (aHR = 2.251, 1.108–4.575), annual missed dose times >5 days of LPV intake (aHR = 1.889, 1.004–3.554); The risk factor of virologic failure in ART-experienced children was missed dose times >5 days (aHR = 2.689, 1.299–5.604) and mother as caregivers for ART administration (aHR = 0.475, 0.238–0.948). However, during long-term treatment, viral suppression rates between ART-naïve and ART-experienced children remained similar. No significant differences were observed in the immune response, treatment-related grade 2–4 events, and abnormal laboratory test results between ART-naïve children and ART-experienced children.

**Conclusion:**

Our research underscores that with consistent, long-term treatment of LPV/r-based regimens, ART-experienced children can achieve therapeutic outcomes comparable to ART-naïve children. It provides crucial insights on LPV/r-based regimens in pediatric HIV treatment, especially in resource-limited settings where high-cost Integrase Strand Transfer Inhibitors (INSTs) are inaccessible. This evidence-based understanding provides an essential addition to the global therapeutic strategies for pediatric HIV treatment.

## 1 Introduction

By 2022, approximately 1.5 million children were living with the human immunodeficiency virus (HIV) worldwide, and more than 130,000 children were newly diagnosed with HIV each year (1). Antiretroviral Therapy (ART) has decreased HIV-related morbidity and mortality and improved survival and quality of life. However, owing to the limited number of suitable formulations, limited access to pediatric ART drugs, regimen palatability, lack of caregiver support, and forgetfulness (2), Antiviral therapy for children continues to be challenging. Compared with 77% of adults aged 15 years and older receiving antiretroviral treatment, only 57% of children aged 0–14 years had access to antiretroviral therapy in 2022 (1). Access to ART drugs for children was generally 7–12 years later than for adults (3). Data on the effectiveness and safety of pediatric antiretroviral regimens in the long-term treatment of children with HIV-1 were primarily based on extrapolation from adult comparative trials; however, there were limited studies on children. Thus, it was essential for clinicians to assess safe and effective pediatric formulations of antiretroviral regimens for HIV treatment and prevention.

Recently, Integrase Strand Transfer Inhibitors (INSTIs) have emerged as the recommended first-line treatment due to their superior efficacy and safety profiles. However, in resource-limited regions, access to these cutting-edge agents is hindered. Thus, Lopinavir/ritonavir (LPV/r), a boosted protease inhibitor combined with Lopinavir and Ritonavir, maintains its pivotal role as the principal regimen, continuing to be indispensable for pediatric HIV management. Based on a robust inhibitory effect in virus replication (4) and a high resistance gene barrier (5, 6), the World Health Organization (WHO) recommended LPV/r with two nucleoside/nucleotide reverse transcriptase inhibitors (NRTIs) as first-line antiretroviral therapy for all infants and children >14 days and <3 years of age since 2013 (7). In 2021, the Department of Health and Human Services (DHHS) recommended LPV/r combined with two NRTIs as preferred initial regimens for Newborns aged ≥14 days to <4 weeks (8). In China, a free antiretroviral therapy program was initiated nationally in 2006, and LPV/r was accessible freely to all children in 2008. By the end of 2015, China recommended LPV/r-based regimens as first-line antiretroviral therapy to all children aged <3 years. Currently, LPV/r is widely used in China and in resource-limited settings for children.

These studies from IMPACT P1060/P1030 have confirmed the superior virologic outcomes over long-term follow-up of LPV/r-based regimen compared with nevirapine (NVP) in infants and children (9–12), especially among children with prior exposure to single-dose NVP. Therefore, the data advocate for LPV/r as a more effective and safer initial therapy for this population. However, a recurring limitation across these studies is the constrained sample sizes and relatively brief follow-up durations, factors that may affect the generalizability and long-term applicability of the findings.

Here, we conducted a comprehensive, long-term, prospective cohort analysis across multiple medical centers, focusing on both ART-naive and ART-experienced children with HIV-1 infections. The objective was to furnish long-term, real-world data regarding the effectiveness and safety of LPV/r-based antiretroviral regimens. This approach aims to fill existing gaps in the context of sustained therapeutic outcomes and safety profiles, thereby contributing to a more nuanced understanding of LPV/r’s role in pediatric HIV treatment of long-term follow-up.

## 2 Methods

### 2.1 Study design

This was a prospective, open-label, multicenter, long-term cohort study. The study was conducted at 8 sites of HIV/AIDS Clinical Research Centers from 6 provinces in China (Henan, Xinjiang, Guangdong, Guangxi, Yunnan, and Hunan).

### 2.2 Ethies statement

The study was conducted in compliance with local regulatory requirements and was approved by local ethics committees or institutional review boards under the 2008 Declaration of Helsinki (037-002). Written informed consent was obtained from all participants’ parents (relatives or public welfare organizations) before study initiation. This process was conducted by research physicians who provided comprehensive explanations about the study trial, its objectives, potential risks, and benefits. The consent was obtained before any study-specific procedures were initiated, ensuring that the guardians or representatives fully understood the scope of the study.

### 2.3 Subjects

Eligible Participants were children aged 1–12 years (excluding infants under 1 year of age), with documented HIV-1 infection, who had received at least 6 months of treatment containing 2 NRTIs plus LPV/r. All participants were LPV/r-naïve. The children were stratified based on prior antiretroviral treatment (Cohort1: ART-naïve children, and Cohort2: ART-experienced children) at the time of enrollment in this study. Within this cohort, ‘ART-naïve’ children refer to those newly diagnosed with HIV infection, initiating treatment with an LPV/r-based regimen and continuing it for more than 6 months. ‘ART-experienced’ children are defined as those who, following the failure of their previous antiretroviral therapy regimen, switched to an LPV/r-based regimen and maintained it for over 6 months. The exclusion criteria were acute opportunistic infection or organ dysfunction period within the previous 14 days, severe mental and neurological diseases, and alcohol or drug use. Participants with missing baseline CD4 counts and viral loads were excluded.

### 2.4 Procedures

Children with a body weight of 7 to 15 kg were administered a dose of 12 mg LPV plus 3 mg ritonavir per kilogram of body weight twice daily. Children with a body weight of 15 to 40 kg were administered a dose of 10 mg LPV plus 2.5 mg ritonavir per kilogram of body weight twice daily. Children were evaluated every month for the first 3 months, followed by every 3 months thereafter. At each of these visits, a thorough physical examination and routine laboratory tests were performed, including complete blood count, alanine aminotransferase, aspartate aminotransferase, total bilirubin, albumin, globulin, creatinine, urea nitrogen, fasting blood glucose, amylase, triglycerides, and total cholesterol. The HIV-1 RNA viral load and CD4 + T cell count was systematically evaluated every 12 months. If the results indicated antiretroviral treatment failure, subsequent drug resistance testing was initiated for the patients concerned. The method for drug-resistant testing has been described in our previous study (13).

### 2.5 Outcomes

The effectiveness outcome was the incidence of participants with confirmed virologic failure (VF), defined as at least two consecutive measurements of VL > 200 copies/mL after 6 months of ART; the proportion of immune response at months 6, 12, 24, 48, 72, 96, 120, and 156 (defined as CD4% recovered to more than 25% after 12 months of treatment); the incidence rate of poor immune reconstitution (CD4 count <500 and CD4% <25% in children after viral suppression lasting more than 5 years) (14–16); self-reported adherence (measured by caregiver report of any missed doses since the last visit); lost to follow-up (defined as lost to follow-up ≥3 times at clinic visits); death. Safety outcomes included the incidence and severity of adverse events and laboratory abnormalities. Adverse events were graded according to the Division of AIDS grading scale (17).

### 2.6 Statistical analysis

The Chi-square test and Fisher’s exact test were used to evaluate mortality, loss to follow-up, drug resistance, and LPV/r related adverse events, respectively, with a 2-tailed *p*-value of 0.05. Comparisons of missed doses between the groups were performed using the Wilcoxon rank sum test. The probabilities of virological failure between the different groups were estimated via the Kaplan–Meier curve and log-rank test. Proportional hazards Cox regression analysis was used to quantify the risk factors for virologic failure. Risk factors of interest included age at ART initiation (6–10 vs. 1–5), race (Han vs. others), WHO stage (III/IV vs. I/II), baseline CD4% (≥15% vs. <15%), baseline plasma HIV-1 RNA levels (≥50,000 vs. <50,000 copies/mL), the time from diagnosis to treatment (>1 vs. = 1 year), caregivers of ART administration (mother vs. others), clinic setting (rural vs. urban hospital), drug regimen (ABC vs. AZT), and number of missed doses each year (≥5, <5 vs. 0 days). All statistical analyses were performed using SPSS version 26.0 (IBM Corp, Armonk, NY, United States) and GraphPad Prism version 8.0.2 (GraphPad Software, San Diego, CA, United States).

## 3 Results

### 3.1 Baseline characteristics

**Figure 1:**
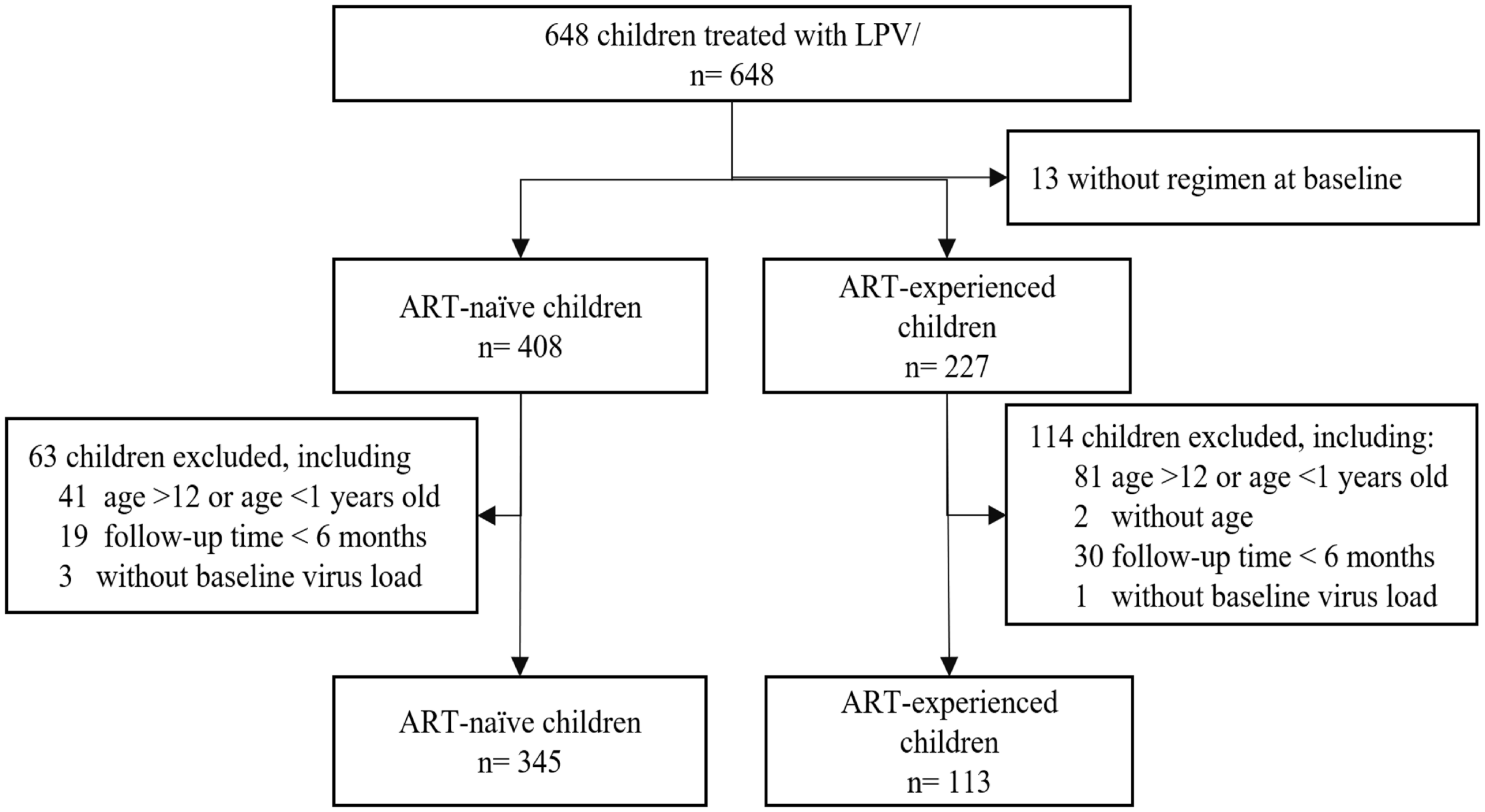
Subjects disposition of the study. From January 2008 to September 2021, 648 children treated with LPV/r were recruited. A total of 458 children were included in the analysis, including 345 ART-naïve and 113 ART-experienced children previously treated.

From January 2008 to September 2021, 648 children treated with LPV/r were recruited. The 13 children excluded from the study were indeed those for whom the baseline treatment regimen was not clearly documented or known. Additionally, in the subgroup of 408 ART-naïve (antiretroviral therapy-naïve) children, we excluded 41 children aged >12 or <1 years old, 19 children were followed up <6 months, and 3 children had missing baseline virus load. In the subgroup of 227 ART-experienced children, we further excluded 81 children aged >12 or <1 year old, 2 children did not have age, 30 children were followed up for <6 months, and 1 child missing baseline virus load. After these exclusions, a total of 458 children were included in the analysis, comprising 345 ART-naïve children and 113 ART-experienced children previously treated (Figure 1).

Among the ART-naïve children, 55.1% (190/345) were male, with a median age of 6.0 years (IQR, 4–9 years), and 29.8% (103/345) had WHO stage III/IV. At baseline, 50.1% (173/345) children had CD4% < 15. ART-naïve children accepted treatment predominantly in city hospitals (85.5%) with a regimen of AZT + 3TC + LPV/r (64.9%).

Among ART-experienced children, most of them were male (63.7%, 72/113), with a median age of 9.0 years (IQR, 6–11 years), and 24.8% (28/113) children had WHO stage III/IV. Approximately 36.3% (41/113) of children had baseline CD4% < 15. ART-experienced children were also treated primarily in city hospitals (68.1%) but used mainly with ABC+ 3TC + LPV/r (60.2%). More detailed baseline characteristics of the ART-naïve and the ART-experienced groups were presented in Table 1.

**Table 1:** Baseline characteristics of HIV-1-infected children initiating LPV/r-based regimen.

		Total *N* = 458	ART-naïve children *N* = 345	ART-experienced children *N* = 113
Age at LPV/r initiation, *n* (%)	1–6	210 (45.9)	179 (51.9)	31 (27.4)
	7–12	248 (54.1)	166 (48.1)	82 (72.6)
	Median (IQR), years	7 (4, 10)	6 (4, 9)	9 (6, 11)
Sex, *n* (%)	Female	196 (42.8)	155 (44.9)	41 (36.3)
	Male	262 (57.2)	190 (55.1)	72 (63.7)
Race, *n* (%)	Han	334 (72.9)	242 (70.1)	92 (81.4)
	Others	124 (27.1)	103 (29.9)	21 (18.6)
WHO stage, *n* (%)	I/II	327 (71.4)	242 (70.1)	85 (75.2)
	III/IV	131 (28.6)	103 (29.8)	28 (24.8)
Date at enter the study	2008–2011	209 (45.6)	118 (34.2)	91 (80.5)
	2013–2014	227 (49.6)	227 (49.6)	0
	2020–2021	22 (4.8)	0	22 (19.5)
Baseline CD4, *n* (%)	<15	214 (46.7)	173 (50.1)	41 (36.3)
	≥15 to <25	139 (30.3)	105 (30.4)	34 (30.1)
	≥25	105 (22.9)	67 (19.4)	38 (33.6)
Baseline CD4 count	Mean (SD), cells/mm3	506.37 (441.75)	491.15 (437.21)	553.29 (454.24)
	Median (IQR), cells/mm3	416 (211, 688)	398 (194, 663)	451 (259,748)
Baseline CD4/CD8 ratio	Mean (SD)	0.45 (1.49)	0.44 (1.62)	0.46 (0.73)
	Median (IQR)	0.30 (0.14, 0.51)	0.29 (0.14, 0.50)	0.33 (0.15, 0.57)
Baseline viral load, *n* (%)	Median (IQR), copies/mL	24,843 (4,877, 121,500)	30,000 (6,115, 150,000)	15,000 (915, 63,050)
	Not Test	35 (7.6)	35 (10.1)	0 (0)
Treatment center, *n* (%)	City hospital	372 (81.2)	295 (85.5)	77 (68.1)
	Rural hospital	86 (18.8)	50 (14.5)	36 (31.9)
Caregiver for ART administration, *n* (%)	Mother	225 (49.1)	168 (48.7)	57 (50.4)
	Others	231 (50.4)	176 (51)	55 (48.7)
	Missing data	2 (0.4)	1 (0.3)	1 (0.9)
ART regimen, *n* (%)	AZT + 3TC + LPV/r	235 (51.3)	224 (64.9)	11 (9.7)
	ABC+ 3TC + LPV/r	179 (39.1)	111 (32.2)	68 (60.2)
	Others	44 (9.6)	10 (2.9)	34 (30.1)

IQR, interquartile range.

### 3.2 Subject disposition

The median follow-up time was 7.3 (IQR 5.5–10.5) years, and the longest follow-up time was 13.3 years. The total follow-up time was 3264.3 person-years, including 2,441 person-years (*n* = 345) in ART-naïve children and 823.3 person-years (*n* = 113) in ART-experienced children. Of the 458 subjects who entered the study, 21.2% (97/458) were lost to follow-up. The rate of loss to follow-up was 22.6% (78/345) in the ART-naïve children and 16.8% (19/113) in the ART-experienced children. No differences were observed between the ART-naïve children and ART-experienced children (*p* > 0.05).

Sixteen children died, with a mortality of 0.5 per 100 person-year (95% CI 0.3–0.8). Six patients died of AIDS-related diseases, including tuberculosis, lobular pneumonia, diarrhea, fungal pneumonia, and cytomegalovirus enteritis. Two patients died of non-AIDS-related diseases. The causes of death included measles and hand-foot-and-mouth disease. Eight patients died of unknown causes. No difference in mortality was observed between ART-naïve and ART-experienced children (*p* > 0.05) (Supplemental Table S1). Five children were transferred to other treatment centers and ended the study. Two children changed regimens due to adverse reactions, including vomiting and diarrhea, and one child changed regimens due to tuberculosis.

### 3.3 Virological responses

**Figure 2:**
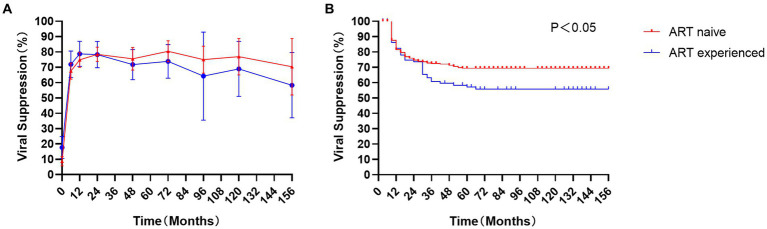
Virological response in children: The proportion of viral suppression **(A)** and Kaplan Meyer (KM) analysis **(B)** in ART-naïve children (red) and 113 ART-experienced children (blue).

After 6, 12, and 24 months of treatment, approximately 67.5 %, 74.8%, and 78.5% of ART-naïve children achieved viral suppression (defined as HIV-1 RNA < 50 copies/mL), where 72.0%, 78.8%, and 78.3% in ART-experience children, respectively (Figure 2A). However, there was no significant difference in the rate of viral suppression between ART-naïve and ART-experienced children (*p* > 0.05) throughout the follow-up period. The incidence density of virologic failure (defined as at least two consecutive measurements of VL > 200 copies/mL) was 4.3 (95% CI 3.6–5.0) per 100 person-years, including 4.1 (95% CI 3.3–4.9) in ART-naïve children and 5.0 (95% CI 3.5–6.5) in ART-experienced children. Kaplan Meyer (KM) curve analysis showed that ART-experienced children were at a higher risk of virologic failure than ART-naïve children (*p* < 0.05) (Figure 2B).

Cox regression analysis showed that rural hospitals (aHR = 2.251, 95% CI 1.108–4.575) and missing doses for more than 5 days (aHR = 1.889, 1.004–3.554) were risk factors for predicting virologic failure in ART-naïve children. In ART-experienced children, missed doses >5 days (aHR = 2.689, 1.299–5.604) were important risk factors, while the mother as a caregiver (aHR = 0.475, 0.238–0.948) for ART administration was a protective factor among ART-experienced children (Table 2).

**Table 2:** Characteristics associated with virological failure in human immunodeficiency virus-infected children.

	ART-naïve children				ART-experienced children			
	Non-adjusted model		Adjusted model		Non-adjusted model		Adjusted model	
	HR (95% CI)	*p*-value	aHR (95% CI)	*p*-value	HR (95% CI)	*p*-value	aHR (95% CI)	*p*-value
Age at ART initiation 6–10 (vs. 1–5)	1.746 (1.166–2.613)	0.007	1.29 (0.736–2.261)	0.373	3.765 (1.474–9.615)	0.006	2.244 (0.810–6.220)	0.12
Race Han (vs. other)	1.715 (1.145–2.567)	0.009	1.314 (0.684–2.523)	0.412	1.195 (0.552–2.588)	0.652		
WHO stage III/IV (vs. I/II)	1.117 (0.733–1.703)	0.606			0.823 (0.403–1.681)	0.594		
Entering the study at 2008–2011 (vs. 2013–2014 or 2020–2021)	0.762 (0.511–1.135)	0.181			0.935 (0.450–1.939)	0.856		
Baseline CD4 ≥ 15 (vs. < 15)	1.044 (0.704–1.548)	0.832			0.475 (0.257–0.879)	0.018	0.682 (0.350–1.328)	0.26
Baseline Viral Load ≥100,000 (vs. < 100,000)	0.964 (0.636–1.459)	0.861			1.891 (0.965–3.709)	0.064		
Time from diagnosis to treatment >1 years (vs. < 1 years)	1.364 (0.913–2.038)	0.13			2.045 (1.072–3.903)	0.03	1.138 (0.545–2.377)	0.731
Caregivers for ART administration-mother (vs. others)	0.678 (0.455–1.012)	0.057	0.814 (0.5–1.325)	0.408	0.503 (0.264–0.960)	0.037	0.475 (0.238–0.948)	0.035
Clinic setting–Rural (vs. Urban)	3.61 (2.427–5.369)	< 0.001	2.251 (1.108–4.575)	0.025	1.054 (0.563–1.976)	0.869		
Regimen – ABC-based regimen (vs. AZT-based regimen)	0.678 (0.427–1.076)	0.099			1.637 (0.499–5.368)	0.416		
Miss doses every year <5 days (vs. 0)	2 (1.005–3.981)	0.048	1.459 (0.695–3.061)	0.318	1.737 (0.648–4.66)	0.272	1.647 (0.598–4.537)	0.334
Miss doses every year ≥5 days (vs. 0)	2.775 (1.592–4.835)	< 0.001	1.889 (1.004–3.554)	0.048	3.077 (1.533–6.176)	0.002	2.698 (1.299–5.604)	0.008

### 3.4 Immune responses

**Figure 3:**
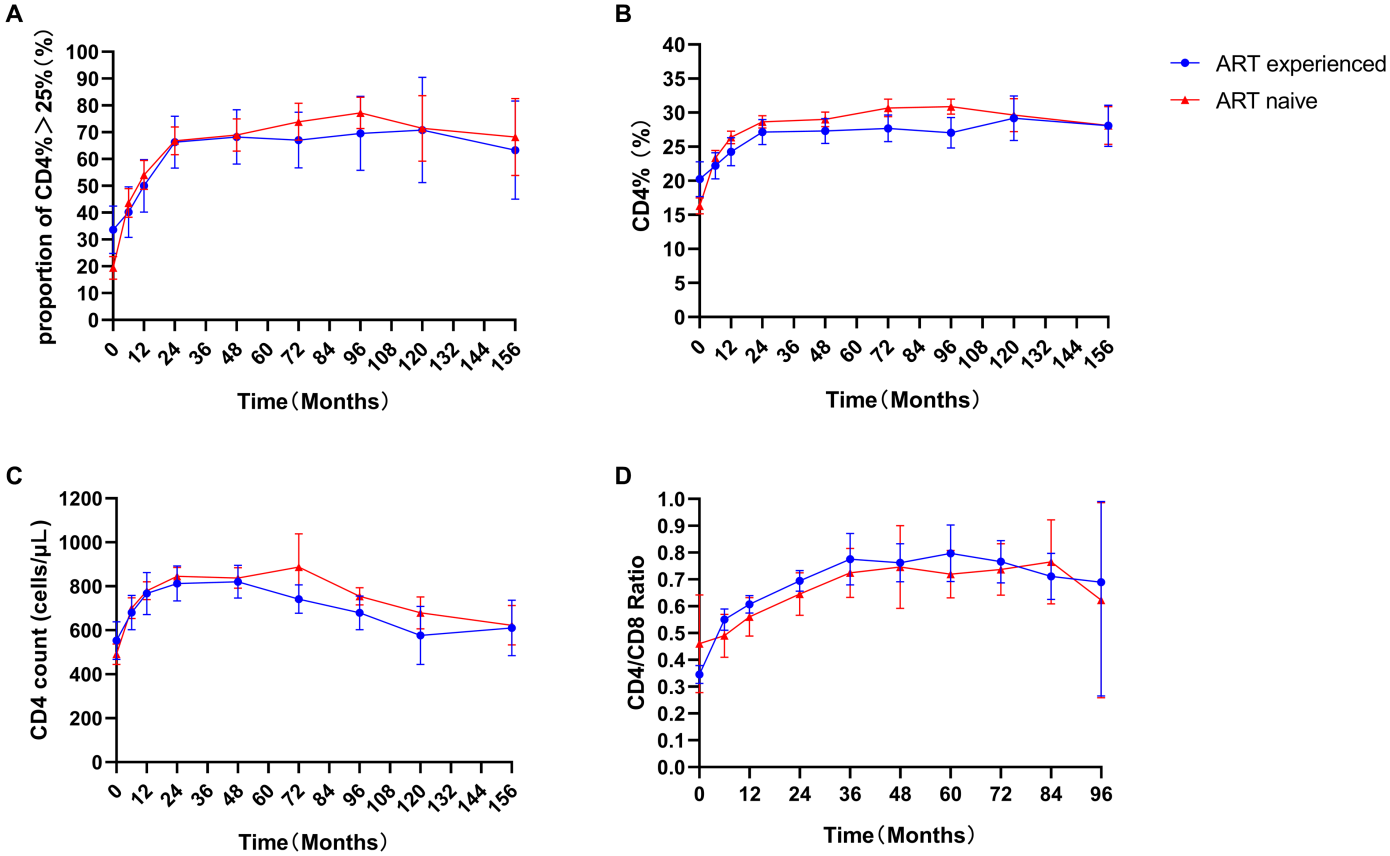
Immune response in children. Proportion of CD4% > 25% **(A)**, CD4% **(B)**, CD4 count **(C)**, and CD4/CD8 ratio **(D)** in ART-naïve (red) and 113 ART-experienced children (blue).

During the follow-up, 53.1%, 66.6%, 68.8%, and 75.8% of all children exhibited a recovery of more than 25% from their baseline CD4 percentages at 12, 24, 48, and 96 months, respectively (Figure 3A). No difference was observed between ART-naïve and ART-experience children (*p*  > 0.05). No significant differences were observed in the CD4 count, CD4%, and CD4/CD8 ratio between the ART-naïve and ART-experienced children (*p* > 0.05) (Figures 3B–D). At ART initiation, children with lower baselines of CD4 count, CD4%, and CD4/CD8 ratio showed greater improvements than those with higher baselines (*p* < 0.05). However, after 36 months, there were no statistically significant differences in the ratio of CD4/CD8 over time (*p* > 0.05) (Supplemental Figure S1C), and after 120 months, there was no significant difference in CD4 count or CD4% over time (*p* > 0.05) (Supplemental Figures S1A,B). After 5 years of treatment, the incidence of poor immune reconstitution in ART-naïve and ART-experienced children was 4.2% (11/261) and 1.1% (1/91), respectively. However, no significant difference was observed between the ART-naïve and ART-experienced children (*p* > 0.05).

### 3.5 Antiretrovirals resistance

At baseline, a total of 363 children had available samples and were successfully sequenced for drug resistance tests. At baseline, all children demonstrated full susceptibility to LPV/r. Among them, ART-naïve children exhibited no resistance to NRTIs or NNRTIs, while 40.4% (36/89) of the ART-experienced children demonstrated drug resistance to either NRTIs or NNRTIs. During follow-up, 203 children had available samples and were successfully sequenced for drug resistance tests. Increased drug resistance was reported in ART-experienced children (10.1% in ART-experienced children vs. 1.8% in ART-naïve children, *p* < 0.022). One ART-naïve child and two ART-experience children showed resistance to LPV/r (Supplemental Table S2).

### 3.6 Medication adherence

Among the caregivers of 458 children, 225 were mothers, 136 were fathers, 71 were other relatives, 24 were from social welfare organizations, and 2 were unknown. The level of adherence, assessed by caregiver reports of any missed doses since the last visit, was obtained from 186 of 345 ART-naïve children and 89 of 113 ART-experienced children (Supplemental Table S3). Similar adherence patterns were observed in ART-naïve and ART-experienced children (*p* > 0.05). 66.7% (124/345) of the ART-naïve children and 66.3% (59/113) of ART-experienced children reported they never miss a dose in the study. Missed doses ≥5 days per year were reported to be 20.4% in ART-naïve children and 22.5% in ART-experienced children.

### 3.7 Safety and tolerability

The overall adverse event rate of LPV/r-based regimen was 67.0% (307/458) (Supplemental Table S4). ART-naïve children experienced more incidence of overall adverse events than ART-experienced children (72.5% [250/345] vs. 50.4% [57/113], *p* < 0.001), especially vomiting (23.8% [82/345] vs. 8.0% [9/113], *p* < 0.001). Other common adverse events included nausea (18.0% [62/345] vs. 12.4% [14/113], *p* > 0.05), hyperlipidemia (6.7% [23/345] vs. 11.5% [13/113], *p* > 0.05), and diarrhea (7.0% [24/345] vs. 6.2% [7/113], *p* > 0.05). Grade 2 to 4 adverse laboratory occurred in 6.1% (21/345) of ART-naïve children and 7.1% (8/113) of the ART-experienced children (*p* > 0.05). Two ART-naïve subjects experienced serious adverse events, including thrombocytopenia and diarrhea. One ART-experienced child had serious adverse events due to pancreatitis. Two ART-naïve children reported adverse events leading to discontinuation due to vomiting and diarrhea.

## 4 Discussion

As a large, multicenter, long-term national cohort study that had never been reported before in China, our findings revealed the effectiveness of LPV/r-based ART in effectively suppressing viral replication in children living with HIV-1. Notably, while ART-experienced children were at a higher risk of virologic failure than ART-naïve children, all children, regardless of prior treatment, recovered to the comparable level of CD4 + T cells and had a low incidence of poor immune reconstitution with LPV/r-based regimen. Our study provides important insights into the long-term effectiveness and safety of LPV/r-based regimens in ART-naïve and ART-experienced children. In resource-limited areas, access to INSTIs-based treatments like DTG remains challenging. The application and implications of LPV/r treatment for pediatric HIV in China are significant and provide an effective and attainable treatment option for addressing pediatric HIV within similar resource-limited settings.

Previous studies on the viral suppression rate of LPV/r-based regimens reported rates ranging from 49 to 71% among children (18–20), and 62–70% among adults in 12 months, respectively (21, 22). However, these studies had limited sample sizes, potentially affecting the generalizability of their findings. The viral suppression rate observed in our study with a larger cohort was 74.8%; this rate not only provides a more representative estimate but also suggests disparities in disease prevalence, possibly indicative of variations in treatment adherence and care quality. The incidence density of virologic failure in our cohort was 4.3 per 100 person-years, including both ART-naïve and ART-experienced children. This rate is notably lower than what has been reported in recent literature, particularly in sub-Saharan Africa, where a systematic review and meta-analysis indicated a virological failure rate of 29% (23), and a retrospective study in South Africa documented a failure rate of 18.7 per 100 person-years (24).

Overall, Children and adolescents faced a higher risk of VF and drug resistance than adults for a variety of reasons, including limited treatment options, the palatability of pediatric drugs, and adherence challenges (25, 26). It was encouraging to note that ART-experienced children using LPV/r as second-line therapy after prior failure still achieved the same level of viral suppression as ART-naïve children in our study. However, we also found that ART-experienced children would experience a higher cumulative risk of virologic failure than ART-naïve children, which reflected ongoing problems with drug resistance and ART adherence. Regarding ART adherence, the Cox regression analysis demonstrated that missed doses were a crucial predictor in both ART-naïve and ART-experienced children. This result was comparable with that of other studies showing that the risk of virologic failure was directly correlated with the number of missed doses of antiretroviral drugs (27). In our study, among the caregivers of 458 children, 225 were mothers, 136 were fathers, 71 were other relatives, 24 were from social welfare organizations, and 2 were unknown. Of note, the results showed that mothers as caregivers, not other relatives or public welfare organizations, would be an essential protective factor to prevent VF in ART-experience children. The MONOD ANRS 12206 cohort showed that fathers as caregivers were risk factors for VF (28). Other studies from resource-limited countries also reported that the age, marital status, and physical health of caregivers were risk factors associated with adherence (29–31). It suggested that there was a direct influence between children’s adherence to antiretroviral therapy and their caregivers. The commitment and involvement of a responsible caregiver were crucial for children to promote better adherence and successful treatment, especially in ART-experience children. Virologic failure and consequent drug resistance were the main challenges of antiviral therapy in children. Drug interruption and discontinuation caused by poor adherence increase the possibility of treatment failure and accelerate the occurrence of drug resistance. Enhanced adherence was an important factor in the successful ART of children to improve their HIV treatment outcomes.

Regarding drug resistance, our previous study has shown that an NNRTIs-based regimen increased the risk of drug resistance mutations more than PI-based regimens in children at ART initiation by multivariate logistic regression analysis (13). In this study, more drug resistance was observed in ART-experienced children than in ART-naïve children during follow-up. Other studies have reported that when children experience virologic failure, previous NRTIs/NNRTIs exposure could lead to the accumulation of resistance mutations, limiting future treatment options and affecting the potential for future viral suppression (32–34). Additionally, among ART-naïve children, the rural clinic setting was another risk factor for VF, which was consistent with our previous study (35). This reflects the contradiction between the limited resources of rural health facilities and the complexity of pediatric treatment, which highlights the necessity of shifting HIV medical service resources from urban clinic settings to rural clinic settings. After 12, 24, 48, and 96 months, there was a 53.1%, 66.7%, 68.8%, and 75.8% probability of remaining on an LPV/r-based regimen with CD4% recovered to more than 25%, respectively. The result was higher than the study on NNRTI-based ART conducted in Thailand, where the probability of reaching target CD4% was 51% by 24 months (36), indicating a superior immune response associated with the LPV/r-based regimen. Notably, beyond the regimen, baseline CD4% emerged as a more pivotal determinant of immune function reconstitution in children (37). Studies from PACTG 219/219C reported that children whose baseline CD4% < 15% did not reach a level > 25% after 5 years of treatment (15). Another Study of LPV/r-based regimens showed that CD4% at baseline had a direct correlation with achieving CD4 > 25% after 48 months of follow-up (38). Of note, if follow-up was extended to more than 10 years, CD4% reached the same level in our study among children with different baseline CD4% levels. This finding highlights the significance of long-term adherence to antiviral therapy in children.

In both adults and children with HIV, immune reconstitution varies significantly. Poor immune reconstitution was common in adults with HIV-1 but rare in children, accounting for only 4.2% and 1.1% of ART-naïve and ART-experienced children after 5 years in our study, respectively. A study on Children and Adolescents showed that 8% of children did not recover CD4 count >500 after 2 years of therapy (16). This disparity between adults and children might be attributed to the residual thymus function in children and adolescents (39–41). Additionally, in HIV-infected children, the immune response is notably influenced by Natural Killer (NK) cells. The presence of Bw4 and low HLA-A expression alleles in children is strongly linked to enhanced immunological and viral control. In contrast, adults predominantly exhibit HLA-B-mediated T-cell activity (42), which might not be as effective in controlling HIV as the NK cell response in children.

Due to the complexity involved in measuring adherence, various methods have been developed, each exhibiting its own unique strengths and limitations (27, 43). Pill counts provide a direct measure of medication consumption, but they proved logistically challenging and may not accurately reflect actual ingestion (44). Self-reports, while useful in some contexts, are not feasible for children who cannot reliably self-report. Drug concentration measurement in plasma or urine offers an objective measure but requires laboratory resources and may not be widely accessible. Given these considerations, we used the caregiver reports as our primary method for adherence assessment. Despite the potential for underreporting, caregiver reports are practical and generally reliable indicators of true adherence, especially when positive. They allow for the collection of adherence data in real-world clinical settings, which is essential for understanding treatment outcomes in a pediatric population.

The safety data for LPV/r during the randomized phase of our study were consistent with those of previous studies (4, 45–47). In our study, ART-naïve children had a higher rate of overall adverse events than ART-experienced children, especially in gastrointestinal reactions. This discrepancy could be because ART-experience children had greater tolerability for LPV/r (4). As reported in the previous studies, gastrointestinal reactions were the most frequent adverse events associated with LPV/r, including vomiting, nausea, and diarrhea (4, 48). However, vomiting or diarrhea leading to discontinuation in our study was uncommon, accounting for 0.4% (2/458), which was consistent with previous reports (48). Hyperlipidemia was another well-known common adverse effect of LPV/r (45, 46), significantly associated with higher fasting total cholesterol, low-density lipoprotein, and triglycerides levels, which were reportedly associated with greater amounts of subcutaneous fat (47).

This study had some limitations. As a prospective cohort study, inherent limitations arise from the possibility of missing data over the long follow-up period, potentially hindering a holistic assessment and interpretation of LPV treatment effectiveness in pediatric HIV. Additionally, adherence to antiviral therapy was a major risk factor for VF. However, this study only included missed doses and caregiver categories to analyze the correlation between adherence and VF. Further studies were needed to explore the influencing factors of adherence to VF.

## 5 Conclusion

Our findings demonstrate the significant effectiveness and safety of LPV/r-based regimens for sustained viral suppression and immune reconstitution in children living with HIV-1. Notably, while the international pediatric treatment guidelines are evolving with the increasing use of INSTs, the high costs associated with these integrase inhibitors make them less accessible in resource-limited settings. Given this, our extensive cohort study on LPV/r offers pivotal insights for resource-limited settings where integrase inhibitor regimens are unattainable, thereby enriching the current global therapeutic strategies.

## Data availability statement

The original contributions presented in the study are included in the article/Supplementary material, further inquiries can be directed to the corresponding author.

## Ethics statement

The studies involving humans were approved by Beijing Ditan Hospital Capital Medical University. The studies were conducted in accordance with the local legislation and institutional requirements. Written informed consent for participation in this study was provided by the participants’ legal guardians/next of kin.

## Author contributions

XLa: Data curation, Investigation, Methodology, Software, Visualization, Writing – original draft, Writing – review & editing. HaZ: Methodology, Project administration, Writing – original draft, Writing – review & editing. LY: Data curation, Writing – review & editing. HoZ: Funding acquisition, Writing – review & editing. QZ: Data curation, Writing – review & editing. HLu: Data curation, Writing – review & editing. YC: Data curation, Writing – review & editing. HLi: Data curation, Writing – review & editing. JC: Data curation, Writing – review & editing. FYe: Data curation, Writing – review & editing. FYu: Methodology, Writing – review & editing. QX: Writing – review & editing. QL: Writing – review & editing. XLi: Writing – review & editing. XY: Writing – review & editing. CY: Writing – review & editing. FZ: Conceptualization, Funding acquisition, Project administration, Writing – review & editing.
