# Supplementary material for: Thirteen-year viral suppression and immunologic recovery of LPV/r-based regimens in pediatric HIV treatment: a multicenter cohort study in resource-constrained settings of China
Source: Front Med (Lausanne). 2023 Dec 22;10:1313734. doi: 10.3389/fmed.2023.1313734 (PMC10771832; doi:10.3389/fmed.2023.1313734)
Supplement: Supplementary file 1 [file Data_Sheet_1.docx]

Supplementary Material

# Supplementary Figures and Tables

# 1.1 Supplementary Tables

**Supplementary Table 1** **Mortality** **of ART-naïve and ART-experienced children**

|  | Number of deaths | Number of cohorts | Total follow-up time | Mortality (per 100 person-year) | 95%CI | | *P*-value |
| --- | --- | --- | --- | --- | --- | --- | --- |
|  |  |  |  |  | Lower | Upper |  |
| Total | 16 | 458 | 3264.25 | 0.490 | 0.288 | 0.797 |  |
| ART-naïve children | 11 | 345 | 2441 | 0.451 | 0.221 | 0.807 | 0.744 |
| ART-experienced children | 5 | 113 | 823.25 | 0.607 | 0.194 | 1.421 |  |

**Supplementary Table 2 Summary of Drug Resistance**

|  | Total N=458 | ART-naïve children  N=345 | ART-experienced children N=113 | *P*-value |
| --- | --- | --- | --- | --- |
| Resistance at baseline | 9.9% (36/363) | 0% (0/274) | 40.4% (36/89) | <0.001 |
| ≥1 NNRTI mutation | 8.3% (30/363) | 0% (0/274) | 33.7% (30/89) |  |
| ≥1 NRTI mutation | 9.9% (36/363) | 0% (0/274) | 40.4% (36/89) |  |
| ≥1 PI mutation | 0%（0/363） | 0% (0/274) | 0% (0/89) |  |
| Resistance during follow-up | 5.4% (11/203) | 1.8% (2/114) | 10.1% (9/89) | 0.022 |
| ≥1 NNRTI mutation | 3.4% (7/203) | 0.9% (1/114) | 6.7% (6/89) |  |
| ≥1 NRTI mutation | 5.4% (11/203) | 1.8% (2/114) | 10.1% (9/89) |  |
| ≥1 PI mutation | 1.5% (3/203) | 0.9% (1/114) | 2.2% (2/89) |  |

**Supplementary Table 3 Missed doses of ART-naïve and ART-experienced children**

|  | All children N=458 | ART-naïve children N=345 | ART-experienced children N=113 | *P*-value |
| --- | --- | --- | --- | --- |
| Missed doses, n (%) | 275 | 186 | 89 |  |
| 0 days | 183(66.5%) | 124(66.7%) | 59(66.3%) |  |
| <5 days | 34(12.4%) | 24(12.9%) | 10(11.2%) | 0.875 |
| ≥5 days | 58(21.1%) | 38(20.4%) | 20(22.5%) |  |
| No data | 183 | 159 | 24 |  |

**Supplementary Table 4: Summary of LPV/r-related adverse events**

|  | All children N=458 | ART-naïve children N=345 | ART-experienced children N=113 | *P*-value |
| --- | --- | --- | --- | --- |
| **All adverse events** | 67.03% (307/458) | 72.46% (250/345) | 50.44% (57/113) | <0.001 |
| Vomiting | 19.86% (91/458) | 23.76% (82/345) | 7.96% (9/113) | <0.001 |
| Nausea | 16.59% (76/458) | 17.97% (62/345) | 12.38% (14/113) | 0.166 |
| Hyperlipidemia | 7.86% (36/458) | 6.66% (23/345) | 11.5% (13/113) | 0.097 |
| Diarrhea | 6.76% (31/458) | 6.95% (24/345) | 6.19% (7/113) | 0.780 |
| Appetite Loss | 4.14% (19/458) | 3.76% (13/345) | 5.3% (6/113) | 0.659 |
| Stomachache | 2.4% (11/458) | 2.89% (10/345) | 0.88% (1/113) | 0.390 |
| Rash | 1.96% (9/458) | 2.02% (7/345) | 1.76% (2/113) | 1.000 |
| Hyperbilirubinemia | 1.31% (6/458) | 1.73% (6/345) | 0% (0/113) | 0.350 |
| Fatigue | 1.09% (5/458) | 0.57% (2/345) | 2.65% (3/113) | 0.187 |
| Dizzy | 1.09% (5/458) | 1.44% (5/345) | 0% (0/113) | 0.444 |
| ALT abnormality | 1.09% (5/458) | 1.44% (5/345) | 0% (0/113) | 0.444 |
| AST abnormality | 0.87% (4/458) | 1.15% (4/345) | 0% (0/113) | 0.576 |
| Headache | 0.65% (3/458) | 0.86% (3/345) | 0% (0/113) | 1.000 |
| Thrombocytopenia | 0.43% (2/458) | 0.28% (1/345) | 0.88% (1/113) | 0.433 |
| Pathoglycemia | 0.21% (1/458) | 0.28% (1/345) | 0% (0/113) | 1.000 |
| Joint pain in the limb | 0.21% (1/458) | 0.28% (1/345) | 0% (0/113) | 1.000 |
| Dyscoimesis | 0.21% (1/458) | 0.28% (1/345) | 0% (0/113) | 1.000 |
| Pancreatitis | 0.21% (1/458) | 0% (0/345) | 0.88% (1/113) | 0.247 |
| **Grade of adverse events** | | | | |
| Grades 2–4 | 6.33% (29/458) | 6.08% (21/345) | 7.07% (8/113) | 0.707 |
| **Serious adverse events** |  |  |  |  |
| pancreatitis | 0.21% (1/458) | 0 | 0.88% (1/113) | 0.247 |
| thrombocytopenia | 0.21% (1/458) | 0.28% (1/345) | 0 | 1.000 |
| diarrhea | 0.21% (1/458) | 0.28% (1/345) | 0 | 1.000 |
| **adverse events leading to discontinue** | | | | |
| Vomiting | 0.21% (1/458) | 0.28% (1/345) | 0 | 1.000 |
| diarrhea | 0.21% (1/458) | 0.28% (1/345) | 0 | 1.000 |

## 1.2 Supplementary Figures


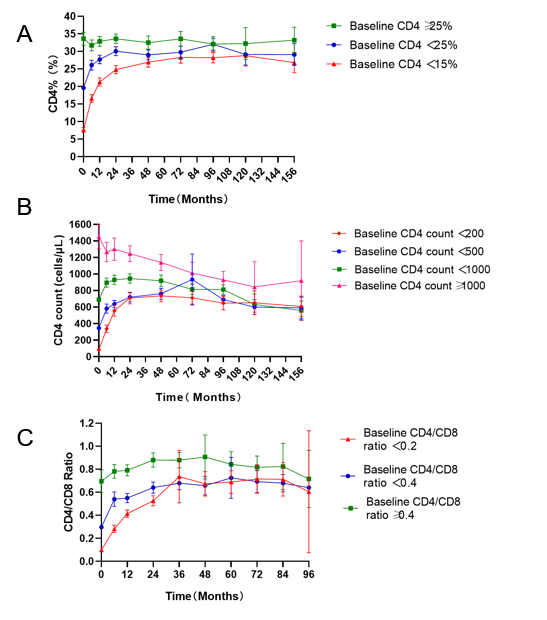


**Supplementary Figure 1: Immune response in** **children with different baselines**

CD4%(Figure 4A), CD4 count(Figure 4B) and CD4/CD8 ratio ((Figure 4C)) in children with different baselines
